# Urban trees as a lever for citizen engagement in public consultation processes: the case of Paris, France

**DOI:** 10.3389/fsoc.2024.1345943

**Published:** 2024-06-05

**Authors:** Amélie Dakouré, Jean-Yves Georges

**Affiliations:** ^1^EVS, CNRS-UMR 5600, Université Jean Moulin Lyon 3, Lyon, France; ^2^Université de Strasbourg, CNRS, IPHC UMR 7178, Strasbourg, France; ^3^Une Fabrique de la Ville, Paris, France

**Keywords:** trees, participatory democracy, governance, ecological urbanism, urban political ecology

## Abstract

In the present context of increasing human population demography, worldwide social crises, and rapid ecological global change, large cities are facing major socio-environmental challenges. This convokes authorities to adapt their governance and urban planning to reconcile urban development, ecological systems, and city dwellers in the most sustainable way. To achieve such goals, local officials have to associate all local actors, including city-dwellers, to the decision-making process through participatory governance and/or participatory systems. Here, we elaborated an original pilot project governance system for a “Participatory System Combining Town Planning and Science” (the 2PS-CiTy), as part of the revision of the Local Urban Plan (LUP) of Paris, France, into a Bioclimatic LUP held from 2020 to 2024. By implementing 2PS-CiTy, we aimed to answer “How to turn trees into a lever for inhabitants’ engagement in urban consultation systems?” Trees were chosen because they are emblematic elements of nature with significant roles in ecosystemic services such as urban climate regulation. Parisians were invited to (i) share in the first questionnaire some information on their knowledge about the LUP and their engagement in it, (ii) identify urban trees they consider remarkable, (iii) explain their choice in a second questionnaire, (iv) contribute to the urban consultation as part of the LUP revision, and finally, (v) give their feedback during a dedicated survey. Out of the 41 Parisians who took part in 2PS-City, 83% declared they were motivated to participate because they could contribute to the tree census, which in turn can constructively contribute to the Parisian LUP revision to bring more nature and sustainability in town. This study demonstrates that trees can be used as a lever for inhabitants’ engagement in urban consultation systems to make cities more sustainable. Our survey also showed that the 2PS-CiTy governance system could be improved by (1) developing a participatory culture among decision-makers and (2) preventing nowadays silo governance from developing the most promising public governance systems that involve the departments of green space, urban planning, and local democracy.

## 1 Introduction

The new Millennium is facing unprecedented climatic, ecological, and social crises, mainly due to unbalanced human demography, natural resource exploitation, and a global economy that has now proven unsustainable (IPBES, 2020). To face this situation, Agenda 21, adopted at the Rio Earth Summit in 1992, sets out several actions for sustainable development in the 21st century. By producing international programs and action plans, international summits organized around solutions to climate change have helped to create international climate governance (Bulkeley and Castán Broto, 2013).

This Agenda 21 devotes an important role to territories. Indeed, it declined at a local scale, so Agenda 21 invites local authorities to put in place local plans that combine the principles of sustainable development with local needs. In September 2015, the 193 Member States of the United Nations adopted the 2030 Agenda for Sustainable Development, thus encouraging an acceleration in the transition of local and regional authorities. By associating the principles of governance with territorial dynamics, using the concept of “territorial governance” makes perfect sense. Indeed, the main objectives of territorial governance include the implementation of projects that aim to develop the territory, target the development trajectories necessary for the territory, or facilitate coordination between territorial actors (Torre, 2023).

### 1.1 An urban political ecology of ecological territorial governance: a theoretical framework

To approach territorial governance at a time of sustainable development agendas, we adopt the *Urban Political Ecology* (UPE) framework. As Rice (2014) argues, UPE is an important framework needed to assess urban climate governance “because of its sustained focus on the city as a socio-ecological process” (Rice, 2014, p. 381). Indeed, Erik Swyngedouw (1996) defined UPE as a field of study that aims to “capture this process of the production of networks and socio-nature to refer to the product, the hybrid, the quasi-object. With this, [he meant] that the ‘world’ is a historical-geographical process of perpetual metabolism in which ‘social’ and ‘natural’ processes combine a historical-geographical ‘production process of socio-nature’ whose outcome (historical nature) embodies chemical, physical, social, economic, political and cultural processes in highly contradictory but inseparable manners” (Swyngedouw, 1996, p. 5).

**Figure 1:**
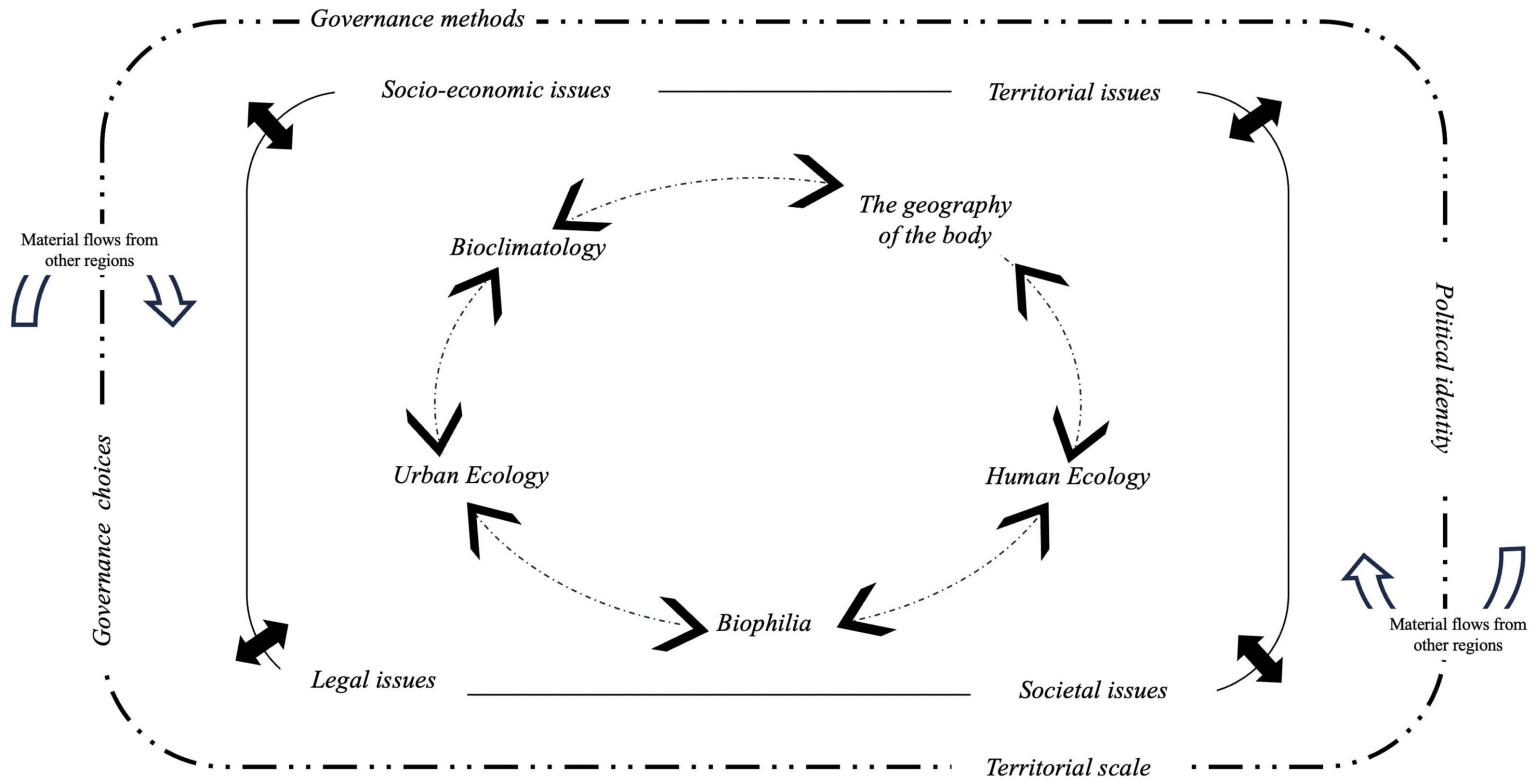
UPE framework to analyze socio-ecological processes regarding political choices and governance frame.

By considering urbanization as an ecological, biological, social, or economic process (Swyngedouw, 1996), UPE integrates into its interpretation of the city many fields of study involving socio-ecological mechanisms and transformations. According to this UPE approach, we selected five fields of study (Figure 1) to provide keys to understanding socio-ecological dynamics in cities. We outline (i) *Human Ecology*, which focuses on the relationship between humans and the (built) environment, considered as an ecosystem, and its components (human and non-human) (Marten, 2001); (ii) *Biophilia*, defined as “the inborn affinity human beings have for other life forms” (Tabb, 2021, p. 3); (iii) (Contemporary) *Urban Ecology*, presented as a discipline that brings together natural resources, species, and the urban environment. It aims to integrate environmental issues into public policy, for instance, in land-use planning (Endlicher et al., 2007); (iv) *Bioclimatology* is a subfield of ecology that studies relationships between living beings (plants, animals, or humans) and the surrounding environment (characterized by physical, chemical, and biological factors) (Paceveaux and Huber, 2007); and (v) *The geography of the body* analyzes the body in space, going beyond a binary way of thinking that separates inside and outside. Some geographers are interested in the body through its adaptation to the geographical environment. Max Sorre, in 1947, linked this approach to the human body as permeable to environmental fluctuations and, by doing so, related it to *Human Ecology* (Di Méo, 2010).

These five fields of study are related to each other and influenced by the economic, political, social, and societal realities of urban areas. Inequalities in access to green spaces in the city reflect this aspect. As Wolch et al. (2014) wrote “Urban green space is also an environmental justice issue, given that in many cities, low- income neighborhoods and communities of color—places where public health challenges tend to be the most critical—often have relatively poor access to safe and well-maintained parks and other types of open space” (Wolch et al., 2014, p. 239).

To highlight interactions between these fields of study and the economic, political, social, and societal urban context, we suggest a UPE theoretical framework (Figure 1).

Based on this UPE theoretical framework (Figure 1), urban municipalities that choose to follow the international climate governance goals must consider their governance methods and political choices by integrating socio-ecological dynamics. To do so, municipalities need specific tools.

### 1.2 The territorial governance tools in France

To meet this challenge, French local authorities may use several tools, including plans and charters.

Since the law of 19 April 2021, experimentation has been added to this toolbox. Under this law, any local authority or group of local authorities may decide, by deliberation, to implement an experiment.

Cities need to take advantage of these new tools to meet several challenges and accelerate the pace of change. Indeed, since the mid-20th century, the surface areas of European cities have increased by 78% (Sainteny, 2008) to the detriment of surrounding natural spaces and city inhabitants’ wellbeing. This situation is expected to increase further since, by 2050, about 70% of the world’s human population is expected to live in cities (United Nation, 2018). Hence, new models of cities and experiments of new urban ecological governance should be envisaged to permit growing numbers of city dwellers to live in the healthiest ways in the city of tomorrow (Rubiera-Morollón and Garrido-Yserte, 2020).

More precisely, since cities are already facing densification issues (Ferrier, 2020) with direct impacts on local climate, environmental and human health (Nieuwenhuijsen et al., 2014), new models of cities and new urban ecological governance experiments, using the UPE framework (Rice, 2014), should be envisaged to permit growing numbers of city dwellers to live in a healthy way in the city of tomorrow (Rubiera-Morollón and Garrido-Yserte, 2020).

Cities, thus, must adapt their urban plans to prevent the deleterious effects of densification on both humans and nature without encroaching on surrounding natural spaces. This highly urgent need prompted the City of Paris (France) to change its urban development strategy by adapting its local urban plan into a *bioclimatic* local urban plan. Novelties included in this new bioclimatic urban plan are aimed at changing the city’s living environment in the most sustainable way for both nature and humans. The city council aims, notably, to strengthen the place of nature in Paris while contributing to connecting people and nature (Ville de Paris, 2022). Better welcoming nature in a densified city is important for improving city dwellers’ experiences (Ferrier, 2020) and wellbeing (Bourdeau-Lepage, 2021).

### 1.3 A human-urban ecology and local democracy diagnosis of cities to identify levers to improve territorial governance

One of the biggest challenges for large cities is both *Human Ecology* and *Urban Ecology*. It is to reconcile ecological development with urban life at large (Francis and Lorimer, 2011). Indeed, nowadays, city inhabitants are increasingly disconnected from nature (Soga and Gaston, 2016; Cox et al., 2017), reducing people’s knowledge and acceptance of biodiversity at large (Cormier et al., 2012). Consequently, conflicts may appear between humans and other species (plants and animals, native and exotic species) since city dwellers prefer to control nature (Dakouré et al., 2020). As an example, people envisage for some—not all—other species a “fair place” as a delimited area (the house, the private garden, or public areas) where other species are exclusively tolerated (Mauz, 2002; Cormier et al., 2012). This “fair place” area tends to extend in city dwellers’ spirit on behalf of the so-called universal right to access space for all species (Cormier et al., 2012) and can lead to massive changes in the urban area’s real estate value by increasing or decreasing housing demands (Hubbard and Brooks, 2021).

A way that may curb the decline in experiences with nature and bring together human and non-human neighbors into a sustainable coexistence in town is to implement original ecological urban governance schemes considering city dwellers’ desire to connect with nature influenced by natural attraction to other species (*Biophilia*).

To better understand public desires and expectations, initiatives have been devised at different scales around the world to improve public participation in decision-making.

Among those initiatives, one may mention deliberative democracy initiatives with the deployment of citizens’ assemblies on the climate crisis, such as the Citizens’ Climate Convention in France in 2019 and the UK Climate Assembly in 2020 (Willis et al., 2022). One can also highlight deliberative initiatives at a local scale, such as the participatory budget in Porto Alegre at the end of the 20th century, which consisted of “an annual cycle of deliberations and decisions concerning the choice of a portion of the municipal funds earmarked for investment, based on local assemblies open to all (…). The projects presented are discussed (…). The projects selected are then submitted to the municipal council” (Garibay, 2015, p.11).

In a different dimension, public participation mechanisms used to assess the social impact of a planning project, such as community forums or key informants (Burdge and Robertson, 1990), are also worth highlighting.

Importantly, local governments are increasingly using citizen participation before drawing up urban plans to best meet the objectives of internationally agreed agendas such as Agenda 21 (Yetano et al., 2010).

In France, municipalities are legally mandated to set up urban local plan designs involving inhabitants in the consultation processes. However, there is a lack of clarity in the definition of participatory processes, even when they are organized at the local level (Nonjon, 2005). For instance, there is no official definition that specifies the modalities of participation, the conditions to be respected, and the expected results. This issue is aggravated by a generalized mistrust in authorities by citizens at both national and local levels, leading to a lack of public interest in local participatory democracy events (Van den Eynde and Orioli, 2009). This is also reflected by the “always the same” phenomenon widely reported by local authorities and experts (France Urbaine and Sciences Po, 2018) and defined as a “difficulty in including certain groups of the population such as young people, the poorer classes, foreigners, households with children and the working population” (France Urbaine and Sciences Po, 2018). Convincing inhabitants to engage themselves in participatory processes related to local urban plans is a real challenge. By addressing links between ecology and democracy, Zask (2023) argues that to improve and strengthen democracy, one should consider culture and habits more than institutions and administrative processes. Thus, one way to encourage public engagement with participatory democracy processes may consist of making such processes more attractive and effectively contribute to people’s wellbeing both in the short and long term. It is, therefore, important to find a way to meet this requirement. Among the determinants of human wellbeing and health, biodiversity and climate have emerged as essential and interconnected elements (IPBES, 2020; Bourdeau-Lepage, 2021).

### 1.4 Creating an ecological territorial governance based on environmental education and engagement

The link between climate, biodiversity, and human wellbeing and health has been clearly proven (IPBES, 2020). The benefits of ecosystem services (services of regulation, support, provisioning, cultural, etc.) offered by nature on the wellbeing of inhabitants have been demonstrated by Millennium Ecosystem Assessment (2023). As such, urban trees constitute a major element of nature in town, as a living legacy of people’s collective history and strong survivors of human footprint on wild nature and significantly contribute to lower heat islands in towns (IPBES, 2020). Being the subject of militant action to protect them (Lagane, 2019), urban trees appear to be relevant candidates for making city dwellers engage themselves in democratic processes such as the Local Urban Plan production process, which refers here to the classic “urban planning code” consultation, i.e., a participation tool that requires people to be involved in land use planning projects that drastically modify their living environment. This is supported by the commit scale developed by Brault-Labbé and Dubé (2009), who proposed that the commit process involves three stages: (i) the motivational component, which activates the process with an emotional approach through attractiveness, (ii) the behavioral component, which permits stakeholders to actively act according to their motivations, and (iii) the cognitive component which reconciles negative and positive aspects of the process to make it happen.

Environmental education is known for putting into perspective the different modalities of participation and can notably have an emancipatory dimension characterized by mobilization through action (Julliard, 2017). Among different initiatives that act on environmental education and, more specifically, nature education, we focus here on participatory science. One can identify different methods of participatory science used by a wide variety of actors, which is why a fairly broad definition is given: “participatory sciences are defined as forms of scientific knowledge production in which non-scientific-professional actors, whether individuals or groups individuals, participate actively and deliberately” (Fortin-Debart and Girault, 2009). Naturalist participatory science programs have been shown to contribute to (i) bringing together people and nature in the real world with clear positive impacts on individual’s wellbeing (Houllier et al., 2017) and (ii) making people actors of large scientific settings and contributors of the collective knowledge for the sake of nature conservation and sustainability (Couvet and Tessèdre, 2013). Participatory science acts on people’s representation, understanding, and acceptance of their surrounding environment. As such, representation is not only determined by our five major perception senses (touch, sight, hearing, smell, and taste) and by the present moment but also by a combination of individual perception, experience, and knowledge.

To get a detailed view of the mechanisms at work when people get involved in the life of the city around an issue such as preserving nature in the city, we focused on a small public.

We favored working with a small group to examine more closely the representations and perceptions of trees among this small public, and then we identified in the literature that working in small groups would enable us to provide better support (Fung, 2003; Blondiaux, 2008).

### 1.5 The pilot project designed and studied: the small biophilic public in Paris, France

Cities can also set up experiments. Within the revision of the Local Urban Plan (LUP) to the bioclimatic LUP of the city of Paris (France) held in 2022, we elaborated a pilot and exploratory study, which is an original experimental governance system for “Participatory System Combining Town Planning and Science” (hereafter referred as 2PS-CiTy). This first pilot can be reproduced and/or adjusted (e.g., by incorporating areas for improvement) by local authorities wishing to experiment with such a system of governance in their cities. Our original design associates urban participatory processes and participatory sciences with a specific focus on urban trees to test whether the integration of city dwellers’ perception, knowledge, and desire for trees as elements of urban nature and landscapes can upgrade LUP to a sustainable, socially supported bioclimatic LUP. By implementing 2PS-CiTy, we aimed to tackle the following question: How to turn trees into a lever for inhabitants’ engagement in urban consultation systems? As such, we addressed the participants’ perception and representation of trees in their town and the value they give them to highlight how the attractiveness of trees can encourage participants to engage in participatory processes thanks to biophilia. Biophilia is understood here as the innate love of life and all living organisms (Gunderson, 2014), particularly in relation to trees (Vainio et al., 2023). The test of a pilot version of DisPAUS thus seeks to understand whether and how biophilia, in the sense of Fromm (1973), toward trees can be a democratic driving force.

## 2 Materials and methods

### 2.1 Volunteer sampling

In the case of small groups, it is common to observe voluntary self-selection processes (Fung, 2003).

Then, to conduct this pilot and exploratory study, we used the volunteer sampling method (Murairwa, 2015). We wanted to better understand individuals who would choose to participate in this experimental participatory process. More specifically, we aimed to describe their representation and perception of trees. Although the results will not lead to conclusions that can be applied to a larger population, we wanted to find out whether this scheme could help to overcome the “always the same” phenomenon. Thus, we wished to better understand the selection bias induced by this experimentation and determine if 2PS-CiTy could attract people who are not used to getting involved in the urban consultation process. Thus, we use selection bias as an outcome. We also wanted to know whether this scheme could enhance participants’ attention to trees located in their everyday environment.

We considered suitable participants to experience the lives of adult people living in Paris who declared themselves to be receptive and volunteered to join the experience as a long and engaging process. We did not include any elected representatives in our sample. We centered our analysis on inhabitants and their relationship with their private space and their living space (which encompasses the various spaces crossed daily, including some public and shared spaces) in Paris. We did not extend the study to users of public spaces because Paris is a world city hosting a large proportion of people who are not inhabitants, such as workers living in the peri-urban, and tourists.

From April to late September 2022, we used two communication channels to mobilize and solicit participants. First, the temporary proximity channel consisted of meeting people in their daily lives in the town (canvassing in streets and markets) and during neighborhood councils to which we were invited. Second, the virtual proximity was based on virtual tools to reach people (social media—Instagram, Lindlink, Twitter, and Facebook using hashtags of every borough; emails sent by municipalities’ local democracy services, neighbor councils, and social organizations). To obtain a mail list of Parisians keen on engaging in our setting, a registration form was attached to those virtual tools permitting people to share their contact details. The mail list was created for three major purposes: (i) distribute the complete information of the experiment through a PDF document, ensuring candidates were best informed about the process before they actually engage; (ii) distribute a personal data protection and consent form to be signed and returned by the candidate; and (iii) maintain appropriate virtual proximity with the participants through the 2PS-CiTy Instagram account providing dynamic updates, reminders, news, and practical information regarding the degree of advancement of the project. Only candidates who signed the data protection and consent forms were considered participants. In total, 41 Parisians participated.

The recruiting campaign ended after the first public meeting organized by the City Hall as part of the local urban plan review.

Each respondent sent the signed form. We did not have to send a reminder.

### 2.2 The 2PS-CiTy: a 5-stage process based on the commit scale

**Figure 2:**
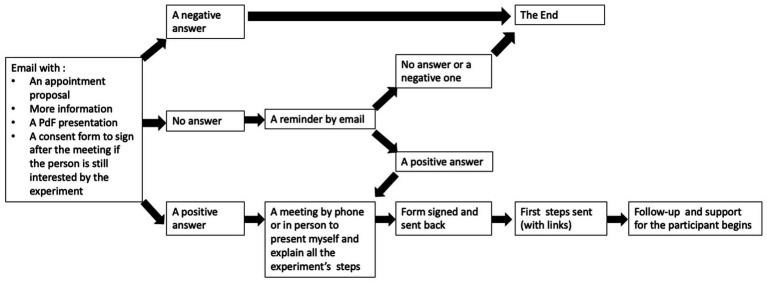
Process flowsheet.

The 2PS-CiTy pilot project was composed of 5 stages (Figure 2). Stage 1 consisted of participants filling in a questionnaire (Appendix 1), entitled “Parisian trees, the local urban plan and you,” aimed at gathering information such as their borough, habits, perception, and representation of trees in their city. Then, participants were asked (stage 2) to (i) identify at least one tree in Paris they considered as remarkable, (ii) take two pictures of that-those trees, and (iii) upload them on the new online platform: Paris tree observatory (CAUE, 2022). These pictures were to be shared with information regarding each tree’s name, location, and surroundings. This participatory platform was chosen for two main reasons: The first one is that it started simultaneously with our pilot project and is easy to handle for novice users as the platform organizers shared a video to explain the functioning of this participatory platform. The second one is that this Paris tree observatory platform contributes to preserving trees because, according to the Council of State’s jurisprudence, a ban on building in an urban area may be allowed if it is aimed at an ecological objective, on condition that the location and delimitation are specified, and its necessity is justified. Thus, to introduce a prohibition on building on a parcel of land to achieve an ecological objective, the tree must be known, named, and listed (Legifrance, 2021, ref. no. 436453). To complete data uploaded on the platform by the participants, contributors were asked (stage 3) to fill out a second questionnaire (Appendix 2), entitled “Tell us about your discovery during the census of trees that you considered as remarkable,” aimed at assessing whether participating in this census encouraged participants to contribute to the consultation engaged by the city as a part of the local urban plan review. If so, stage 4 aimed at making participants choose and take part in one of the four main disposals developed by the city council, namely public meetings, and the online civic tool platform idee.paris (Site IDEE Paris, 2022) participatory workshops in borough or urban walks in their borough. Finally, stage 5 consisted of a 30-min interview with the authors (AD) to assess how participants experienced the whole process of such a participatory system.

We drew on work in the psychology of engagement, and more specifically, we linked this 5-stage process with the commit scale developed by Brault-Labbé and Dubé (2009). As mentioned earlier, according to the authors’ commitment scale, there are three component categories: (i) the motivational component (referred to as the answer to the question “Why am I committing myself”), (ii) the behavioral component (referred to as the answer to the questions “How can I commit to?” and “What behaviors or practices should I modify to properly commit?”), and (iii) the cognitive component (referred to as the answer to the questions “What am I committing for?” and “What are the main goals of this engagement?”). Being interconnected with each other, these three components must be brought together for an engagement to last. Based on this commit scale, we propose that perception and representation are key elements of the motivational component. To illustrate this statement, we adapted the commit scale developed by Brault-Labbé and Dubé (2009) (Table 1). We propose that the motivational component refers to reasons that motivate respondents to participate in the 2PS-CiTy. The behavioral component is composed of our predictions and deductions on what could constrain the engagement (lack of time, for instance) and efforts that participants are making to commit despite constraints (express through “even if” or “despite” in the 2nd column of Table 1). The cognitive component shows goals that participants should be able to perceive at the end of the process (3rd column, Table 1) according to our projections realized by considering both the motivation reasons to commit to the 2PS-CiTy and the main issues of participatory governance identified in most developed governance systems (Shashua-Bar and Hoffman, 2000).

**Table 1:** The 2P.S-CiTy 5-stage process according to commit components.

5-stage process		The motivational component “why am I committing myself”	The behavioral component “How can I commit to?”	The cognitive component “What are the main goals of this commitment”
Stage 1	Questionnaire A *Parisian trees, the local urban plan and you*	Identifying a tree that is remarkable to me motivates me	Even if it takes me some time, participating in this participatory process allows participants to be informed and express themselves; Even though I am not always comfortable with digital devices and platforms, I try to understand and adapt to these tools	To improve knowledge on nature in my town and on participatory processes; I understand the concrete positive effects of having identified a tree for preservation
Stage 2	To identify and photograph a tree			
Stage 3	Questionnaire B *Tell us about your discovery during the census of trees that you considered as remarkable*			
Stage 4	To participate to the consultation process related to the local urban plan	Contributing to the preservation of trees in Paris encourages me to participate in the consultation	Despite a busy personal and professional schedule, I take the time to come to the public meeting or the participatory workshop; Despite the technical nature of the document, I go on the civic tech tool “idee.paris” to give my opinion; Even if it requires time, I want to learn more, and I read the documents relating to the Local Urban Plan; Even though I am not always comfortable with digital devices and platforms, I try to understand and adapt to these tools	I understand the concrete positive effects of having identified a tree for preservation; To listen to the experts’ and decision-makers’ explanations even if there is a disagreement between some participants because it brings elements of understanding and nourishes the dialogue
Stage 5	A 30-min interview with A. Dakouré	Helping researchers explore new green and urban governance solutions by sharing my experience of the 2 PS-CiTy process motivates me	Even if it takes me some time, participating in this participatory process allows me to be informed and express myself	Thanks to the exchanges with the researchers, I could share my feedback for them to improve the 2 PS CiTy. They can share their learnings with me, and I can ask to be notified of publications

To conduct the interviews, a three-part interview guide was drawn up: (1) The first part dealt with the monitoring of the pilot project. We wanted to know the number of stages completed, the reasons for any abandonment, the means used to attend the urban consultation, and the desire to re-engage in a similar scheme. (2) The second part focuses on the participants’ feelings and experiences of the scheme. The idea is to measure the benefits of the scheme for the participants and the obstacles encountered. We wanted to work with them to define the limits of the scheme and how it could be improved. Indeed, the idea behind this approach is to operate based on shared intelligence (Cosson et al., 2017) and to allow debate on the terms of participation in the pilot project (Blondiaux, 2008). The aim of this section was also to find out how their relationship with trees will evolve. (3) The final section dealt with arbitration. The idea was to ask the participants how they would arbitrate between several situations that require arbitration concerning the distribution of space in the city among housing, shops, craftsmen, offices, and green spaces.

We conducted these interviews between November and December 2022, once the urban consultation process supervised by the city had been completed. We prefer face-to-face interviews whenever possible. In this case, the interviews took place in cafés. However, we conducted the interviews by video or telephone if, for practical reasons, the participants preferred a remote interview.

## 3 Results

This study is based on the 41 answers to the first questionnaire and the 34 interviews.

This small public that we identify as biophilic, because of its attraction to trees, is not representative. Indeed, as mentioned, we wanted to find out who would be attracted by a participatory urban project for protecting trees. The sample attracted by tree welfare and the characteristics of this raw sample are a central finding here as urban forestry governance aims at “involve[ing] local residents in both the management and maintenance of new and existing urban trees” (Coleman et al., 2023, p. 13).

We did not sort the participants by drawing lots. This is what distinguishes this small biophilic public from a mini public (Fung, 2003).

This gives us some interesting information about the profiles of the participants. This small biophilic public (*N* = 41) is predominantly female (68%).

The two age groups most represented are 60 to 69 (29%) years and 18 to 29 (27%) years. They are followed by those aged 50–59 (15%) years and over 70 (15%) years. The least represented age groups are 30–39 (7%) years and 40–49 (7%) years.

The three socio-professional categories most represented are managers and higher intellectual professions (51%), retired people (27%), and employees (10%).

### 3.1 The “green situation” of participants’ districts

**Figure 3:**
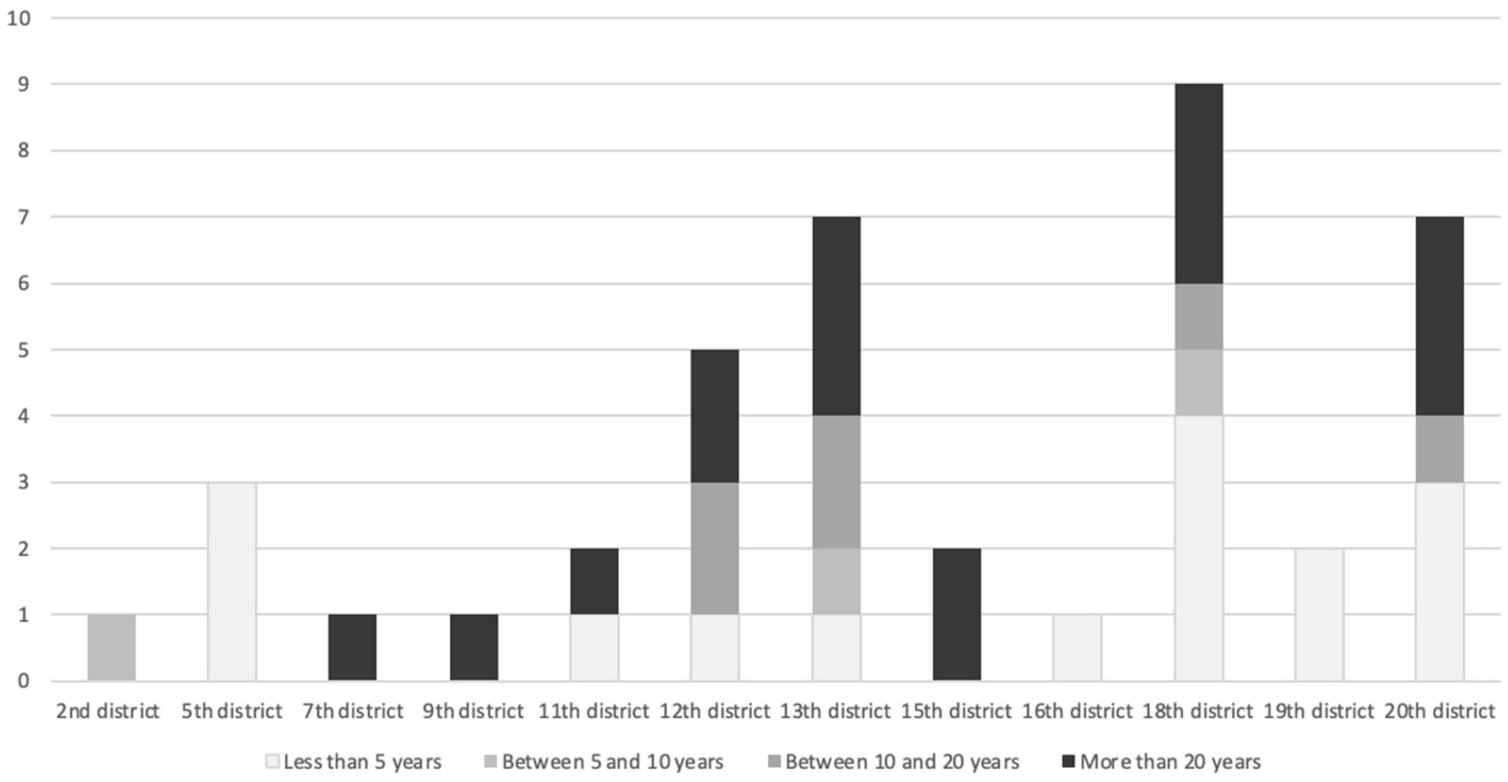
Residence time of the 41 respondents in their current borough.

Participants were living in 12 of the 20 Paris districts (arrondissements). Most participants (16) have either lived in their arrondissement for more than 20 years or for less than 5 years (Figure 3).

**Figure 4:**
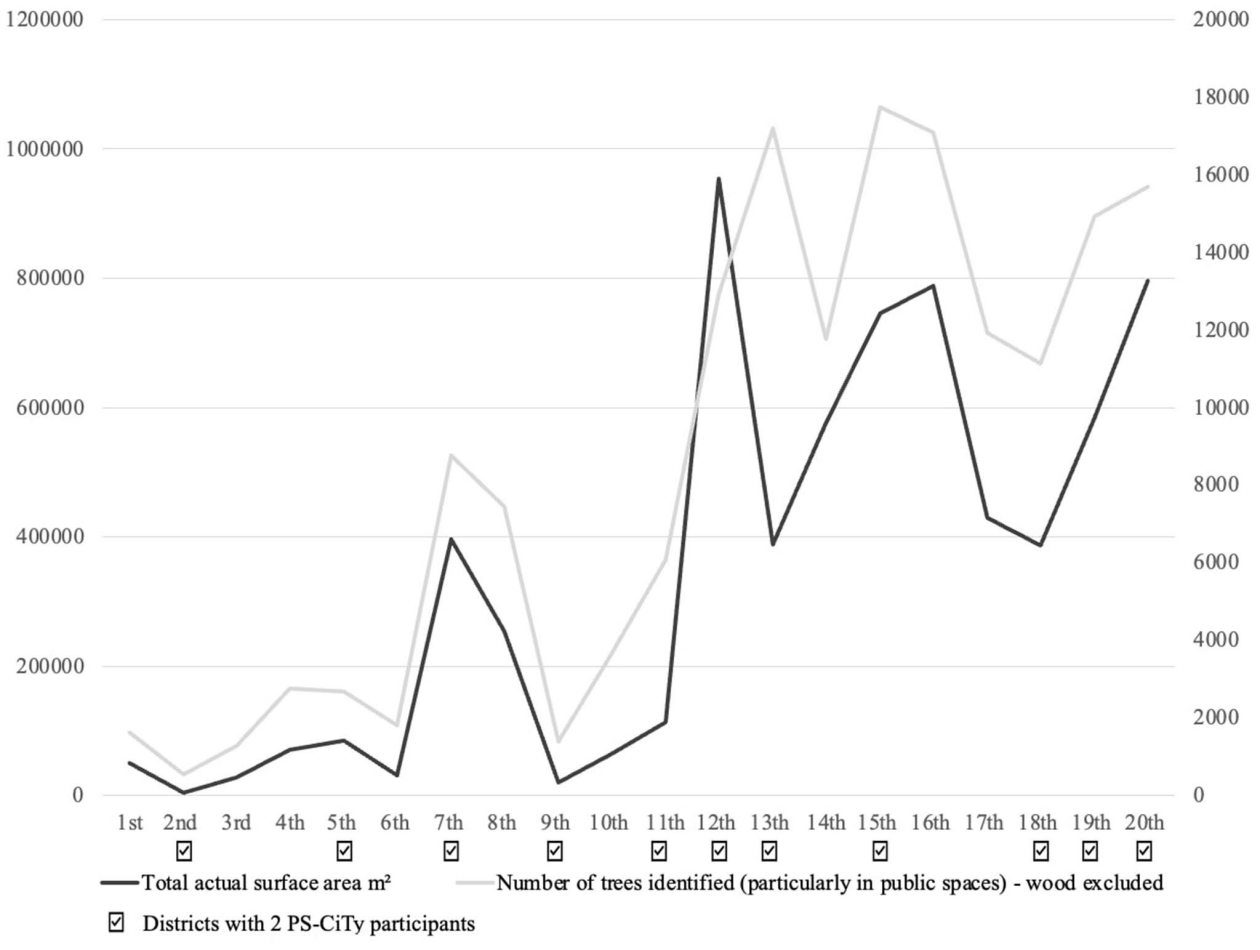
The green situation by district. Source: opendata.paris.fr.

Many of the participants live in the 13th, 18th, and 20th districts, which are on the outskirts of the city and are greener than the one in the heart of Paris (Figure 4).

If we exclude the Bois de Boulogne (16th district) and the Bois de Vincennes (12th district), which increase the real surface area of green spaces in these arrondissements, the participants in the 2 PS-CiTy were living in arrondissements where there are relatively more trees, more green, and related spaces (Figure 4).

### 3.2 Perception and representation of urban trees by respondents

**Figure 5:**
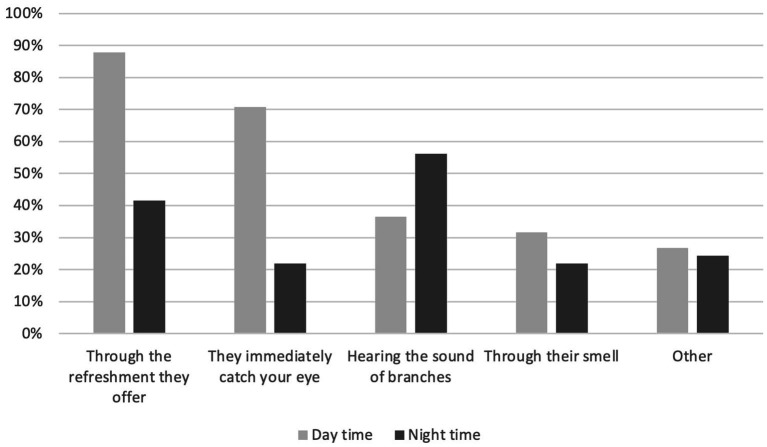
Perception of urban trees during daytime and nighttime for 41 respondents living in Paris, France, 2022.

To the question “What is your perception of urban trees?,” the 41 respondents provided contrasting answers depending on whether they reported daytime or nighttime perception (Figure 5): during daytime, 88% of respondents referred to the refreshment offered by trees and 71% to the visible (sight) element of nature. In contrast, during nighttime, 56% of respondents referred to the sound (hearing) of branches and 41.5% to the refreshment provided by trees.

**Figure 6:**
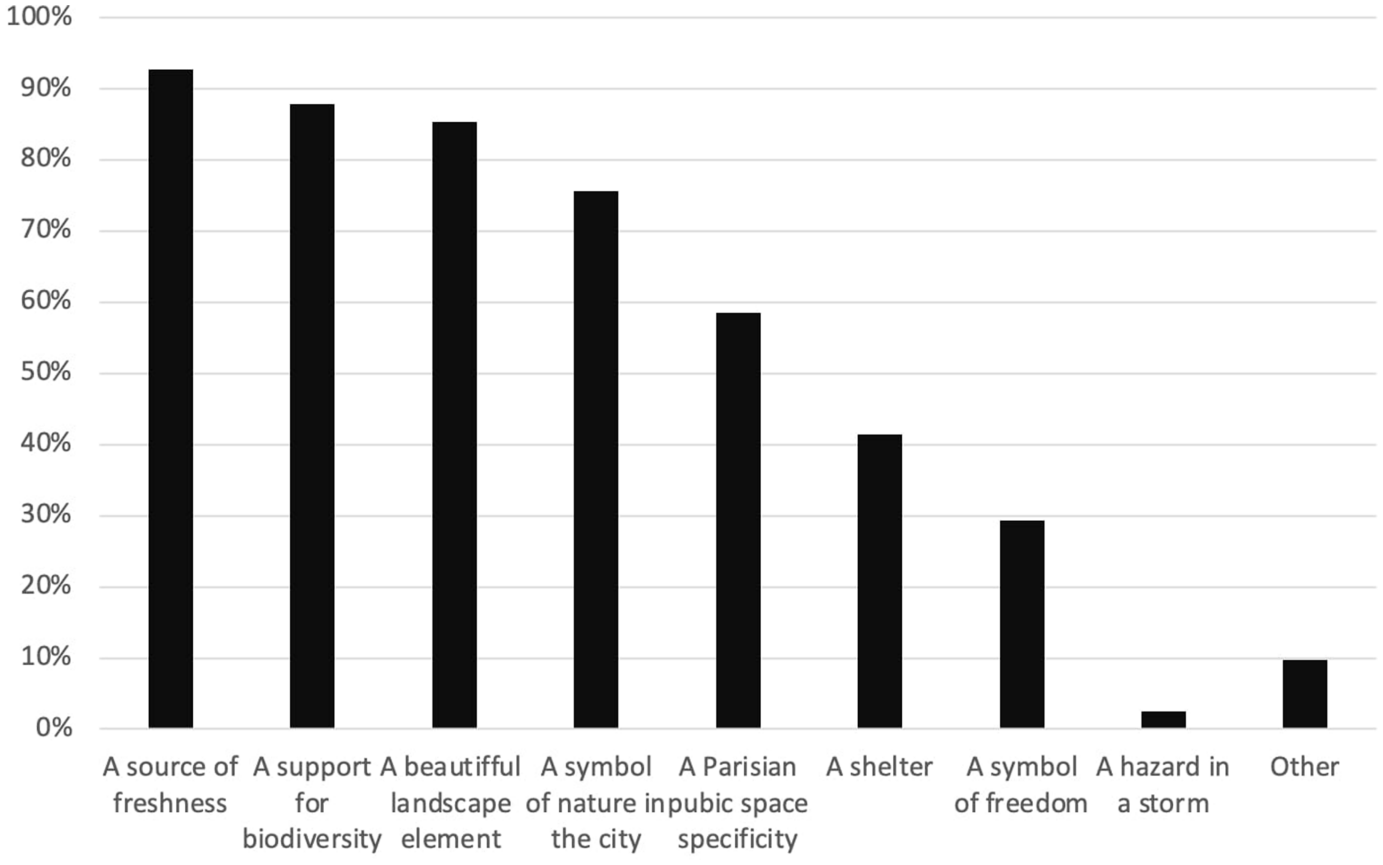
Representations of urban trees for 41 respondents living in Paris, France, 2022.

When it comes to representation (i.e., a more abstract understanding of surrounding items), 93% of participants mentioned trees as a source of freshness, 88% as a support for biodiversity, 85% as an esthetic element of landscape, and 83% as a symbol of nature in the city (Figure 6).

These first results highlight the importance of perception, linked to past experience, in the representation of trees as well as the narrow distinction people make between perception and representation when it comes to thermoception, i.e., the perception of surrounding temperature and thermal fluxes.

### 3.3 Value of urban trees in the living environment of respondents

**Figure 7:**
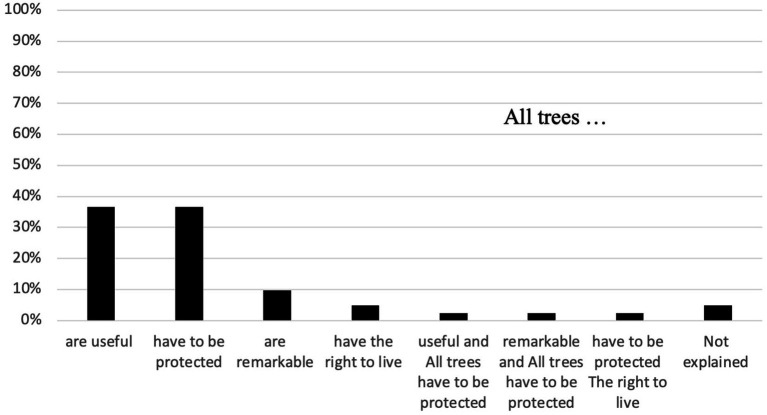
Proportion of the values given to urban trees by 41 respondents inhabiting Paris, France, 2022.

To assess if perception and representation provide any value to urban trees, respondents were asked “*In your opinion, do trees have to be remarkable to be preserved in the city?*” Most of the respondents (95%) gave positive values to urban trees, qualifying them as useful (37%), worth being protected (37%), and remarkable (10%) (Figure 7).

Interestingly, to the question, *“In what situations would you not be ready to accept the presence of a tree near your home?”* 66% of the respondents answered that they would not accept a tree that is not healthy or that represents a risk for humans. To a lesser extent, 20% of the respondents indicated that they are not keen to accept a tree if it prevents light from entering their home, 12% if roots damage public facilities, and 5% if the tree attracts insects or birds such as pigeons or crows.

### 3.4 Levers and obstacles for engagement in 2P.S-CiTy process

**Figure 8:**
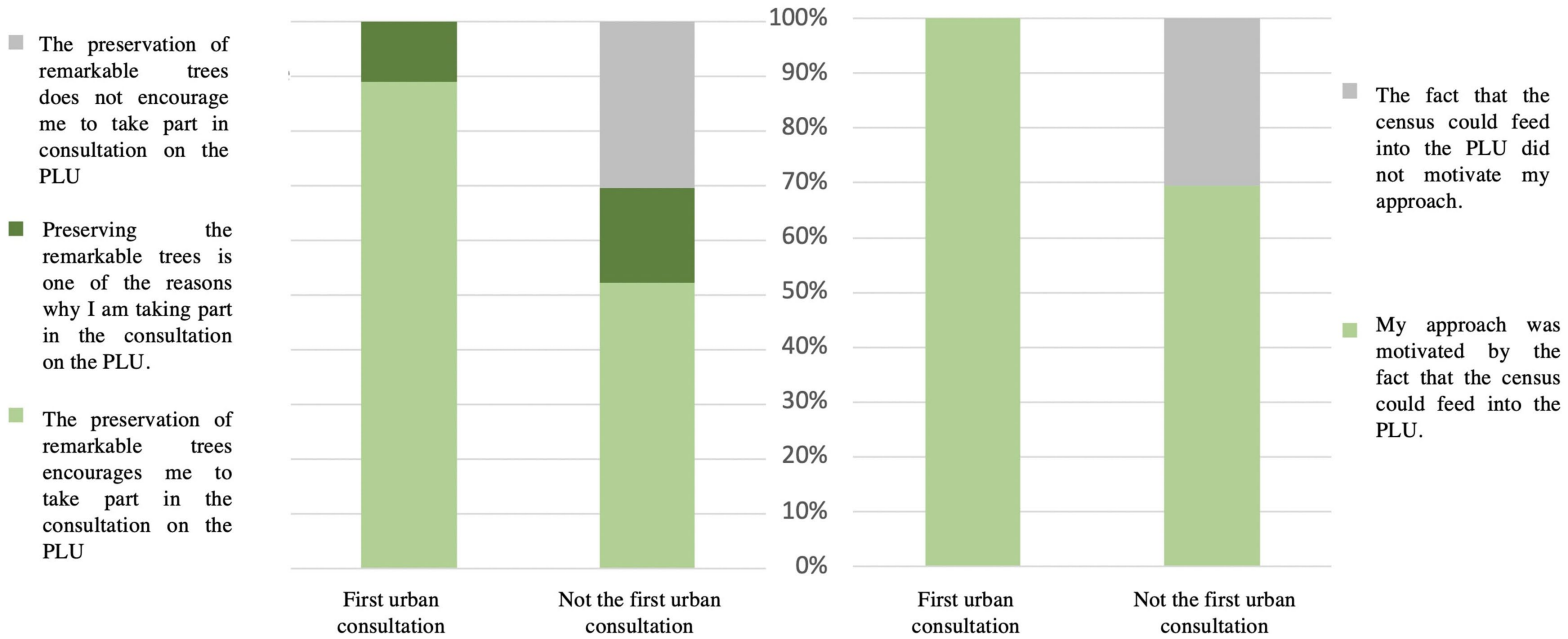
Trees’ preservation as a lever to engage for the first time.

The major reason (83% of the answers) why the 41 respondents were motivated to participate in the 2P.S-CiTy pilot project was because they considered tree censuses can feed the LUP. For 83% of people, integrating the inventory of remarkable trees in the PLUbioclim of Paris is a real motivation to engage in the participatory process, while it is not a reason at all (for the remaining 17%); (Figure 8). Furthermore, it is the reason chosen by the 46% of respondents who contribute to an urban consultation for the first time.

Importantly, most respondents (83%) replied that they were motivated to engage in the 2PS-CiTy pilot project because they considered that tree censuses could contribute to the currently revised Paris Local Urban Plan, and that they were encouraged to take part in the consultation on the Local Urban Plan regulations by and for preserving remarkable trees.

**Figure 9:**
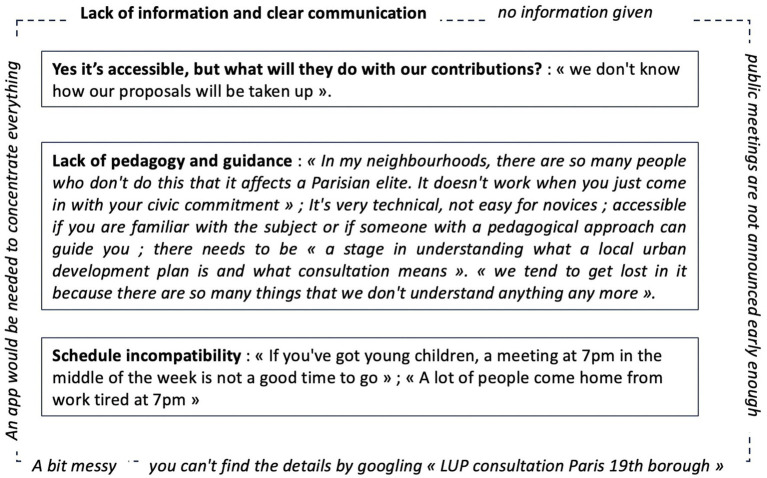
Urban consultation obstacles modeling.

**Figure 10:**
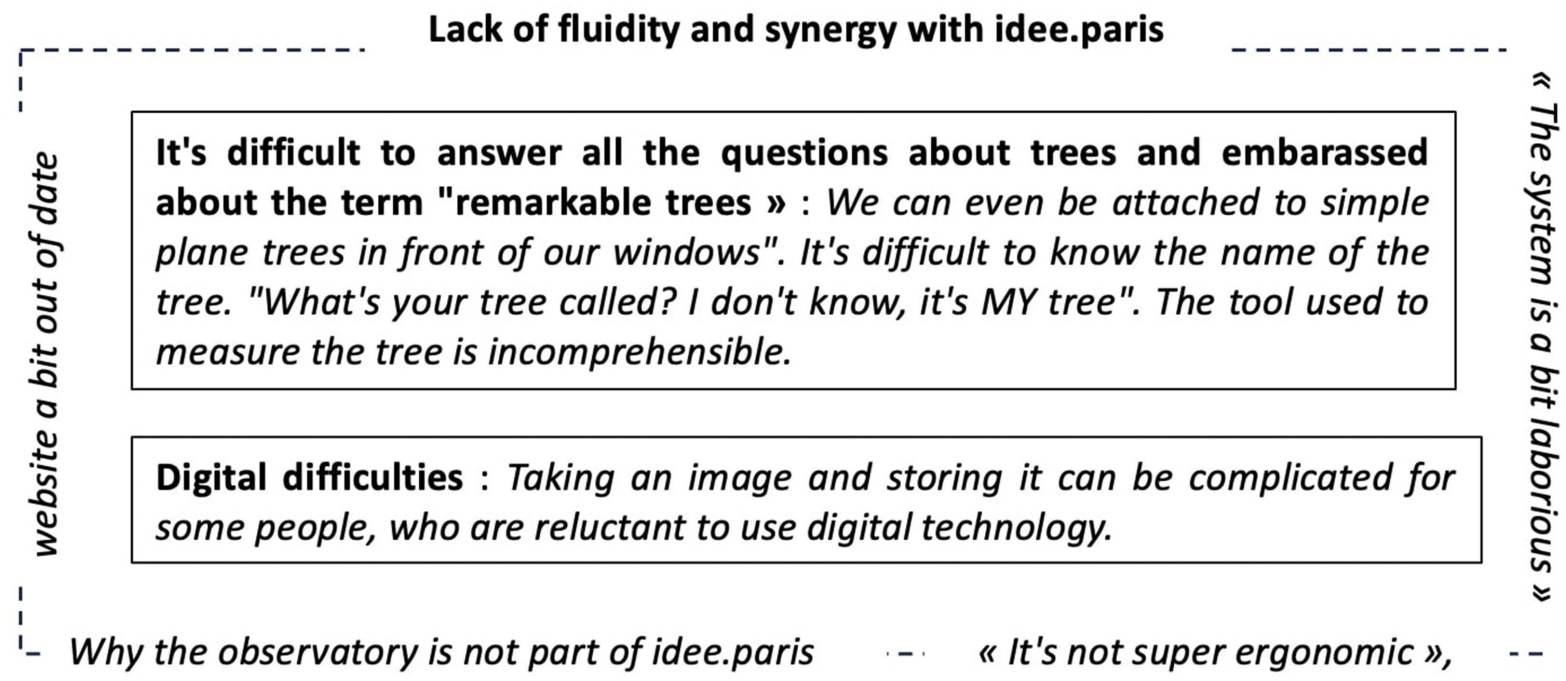
CAUE platform obstacle modeling.

**Figure 11:**
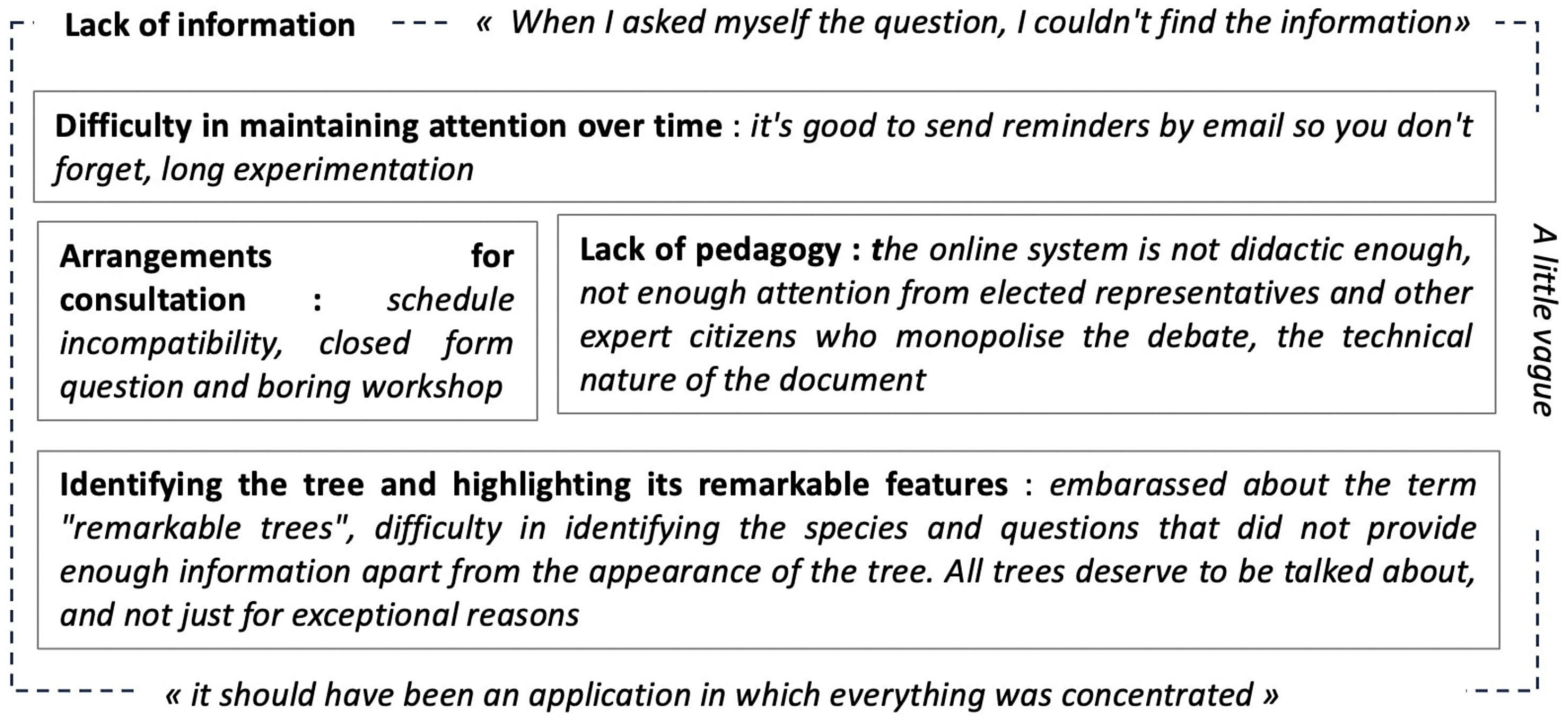
2 PS-CiTy obstacle modeling.

Nevertheless, this lever is held back for some participants. During the interview, three questions were asked of the participants: (1) “Was the consultation set up by the City of Paris accessible?” (Figure 9), (2) “Is the platform developed by the CAUE of Paris to identify trees accessible?” (Figure 10), and (3) “Were you held back by any element of the device?” (Figure 11).

Out of 34 participants who responded to the interview, 26% were held back by nothing. Others gave 5 main reasons for being held back (Figure 11).

Regarding the urban consultation organized by the Paris City Hall, the 2 PS-CiTy participants mentioned four main limits. A transversal and structuring limit that has an impact on others, *the lack of information and clear communication*, and three other limits that weaken the motivational, cognitive, and behavioral components of engagement: (i) A limit that raises suspicions and questions *Yes its accessible, but what will they do with our contributions?* (constrains the cognitive component); (ii) A limit that represents a boundary for understanding the exercise of revising the local urban plan *Lack of pedagogy and guidance* (constrains the motivational and cognitive components); and (iii) A limit linked to the time people can devote to exercising and organizing their *Schedule incompatibility* (constrains the behavioral component) (Figure 9).

Regarding the CAUE platform limits, our participants list three main limits.

Among those limits, one can identify a structuring one called *Lack of fluidity and synergy with idee.paris* (the urban consultation platform) and two limits that constrain the motivational, cognitive, and behavioral components of engagement: (i) *it is difficult to answer all the questions about trees and* [they are] *embarrassed about the term remarkable trees* (constrains the motivational and cognitive components) and (ii) *Digital difficulties* (constrains the behavioral component) (Figure 10).

Regarding the 2 PS-CiTy global pilot project, *Lack of information* is a structuring limit. Four other limits are listed: (i) *Difficulty in maintaining attention over time* (constrains behavioral and cognitive components); (ii) *Arrangements for consultation* (constrains behavioral component); (iii) *Lack of pedagogy* (constrains the motivational and cognitive components); and (iv) *Identifying the tree and highlighting its remarkable features* (constrains the motivational and cognitive components) (Figure 11).

One can see that the limits listed in Figures 6, 7 feed the limits of the 2 PS-CiTy general system.

## 4 Discussion

Our study shows that among the 41 Parisians who participated in the 2PS-CiTy experience, 83% of them considered that contributing to citizen sciences aimed at identifying remarkable trees to preserve them in Paris is a strong motivation to make them engage in this participatory process. It is also the reason mentioned by all new contributors to urban consultation.

We identified this small public as a small biophilic public. We note that this small biophilic public was predominantly made up of women and managers. Moreover, despite the common idea that young people no longer participate in democratic life (Foa and Mounk, 2016), 27% of the sample was under 30 years of age. This is the second most represented age group. The preservation of trees, therefore, attracts the commitment of the young people in our study.

We also noted that this sample was made up of residents from greener boroughs. The relatively smaller number of trees in central boroughs such as the 10th and 3rd may explain why some people were likely discouraged from taking part in the tree census. This interpretation runs counter to what is suggested in the literature, in particular the fact that “if resident satisfaction [concerning the street in which they live and the presence of nature in the area] is low residents are expected to improve the degree of “fit” and also initiate modification to improve well-being and satisfaction” (Coleman et al., 2023, p. 13). Following on from this observation, we could consider that participatory tree planting could then be a solution to improve the satisfaction related to nature in people’s streets. This would encourage residents to protect these new trees by ensuring that they are well-registered. However, numerous barriers to participatory tree planting have also been noted in the literature. These include economic difficulties and lack of time and space (Riedman et al., 2022).

### 4.1 Ecosystem services of trees well perceived by participants

According to respondents’ statements, their relationship with trees is linked to the way they perceive and represent them. As mentioned, during daytime, 88% of respondents referred to the refreshment offered by trees and 71% perceived trees thanks to their sight. During nighttime, 56% of respondents referred to the sound (hearing) of branches and 41.5% to the refreshment provided by trees. Hence, most respondents perceived trees through thermoception (i.e., the perception of surrounding temperature and thermal fluxes). These results support several studies’ statements showing that trees enhance outdoor thermal comfort by reducing urban heat islands, especially in the street canyon (Coutts et al., 2016) (*Bioclimatology*). It also echoes the research work related to the influence of the living environment (light, temperature, etc.) on the body (*Geography of the Body*) (Di Méo, 2010).

This result emphasizes the relevance of ecosystem services provided by trees on respondents’ perception, representation, and wellbeing. Indeed, the present study took place during and right after heavy heat waves that occurred during the summer of 2022. The refreshment provided by trees was, then, sought (regulation ecological service). Figures 3, 4 show a consistency between our respondents’ perception and representation. Thermoception also influenced respondents’ mental representation of trees. Indeed, by classifying answers by ecological services, it appears that trees are mostly perceived and represented through their human/landscaping services. Indeed, 93% of respondents represent trees as a refreshment source, 88% as biodiversity support (*Urban Ecology*), 85% as a beautiful element of the Parisian landscape, and 83% as a symbol of nature (*Biophilia and Human Ecology*) (Figure 4). Human/landscaping ecological services chosen by respondents to describe their representation of trees do not directly reflect their perception. They also represent trees as a symbol of nature and freedom which is formed by relying on social and cultural conventions (Vergote, 1959).

As 83% of respondents considered that preserving trees remarkable for them is a motivation to get involved in 2PS-CiTy, we can deduce that, in this context, respondents’ perception and representation of trees are keys to motivating people to commit to participatory processes. One can wonder if this motivation would remain the same if the representation and perception of trees do not remain positive. Indeed, nature in the city can be perceived as unpleasant when it gets out of hand, takes up unwanted space, or attracts other unwanted species (Wintz, 2019). Therefore, care must be taken to ensure that situations in which respondents do not tolerate the presence of trees are considered. Indeed, respondents indicated few situations in which they would not tolerate the presence of a tree. Most (27 out of 41) of the respondents evoked the trees’ health reasons and the risk it may represent for people if it is unhealthy. Among these 27 respondents, 20 gave this as the only reason. This result suggests that, as the number of trees in private and public places rises, tree surgery plays a relevant role in their acceptance of trees in the city.

As the ecosystem services of trees are well perceived and known by the respondents, they can perceive the purpose of their approach, which is to preserve urban trees and the ecosystem services they provide in the city.

### 4.2 Limits of the 2 PS-CiTy pilot project

Nonetheless, as respondents expressed, this pilot project structure revealed some limits that should be considered to improve the 2 PS-CiTy design.

**Figure 12:**
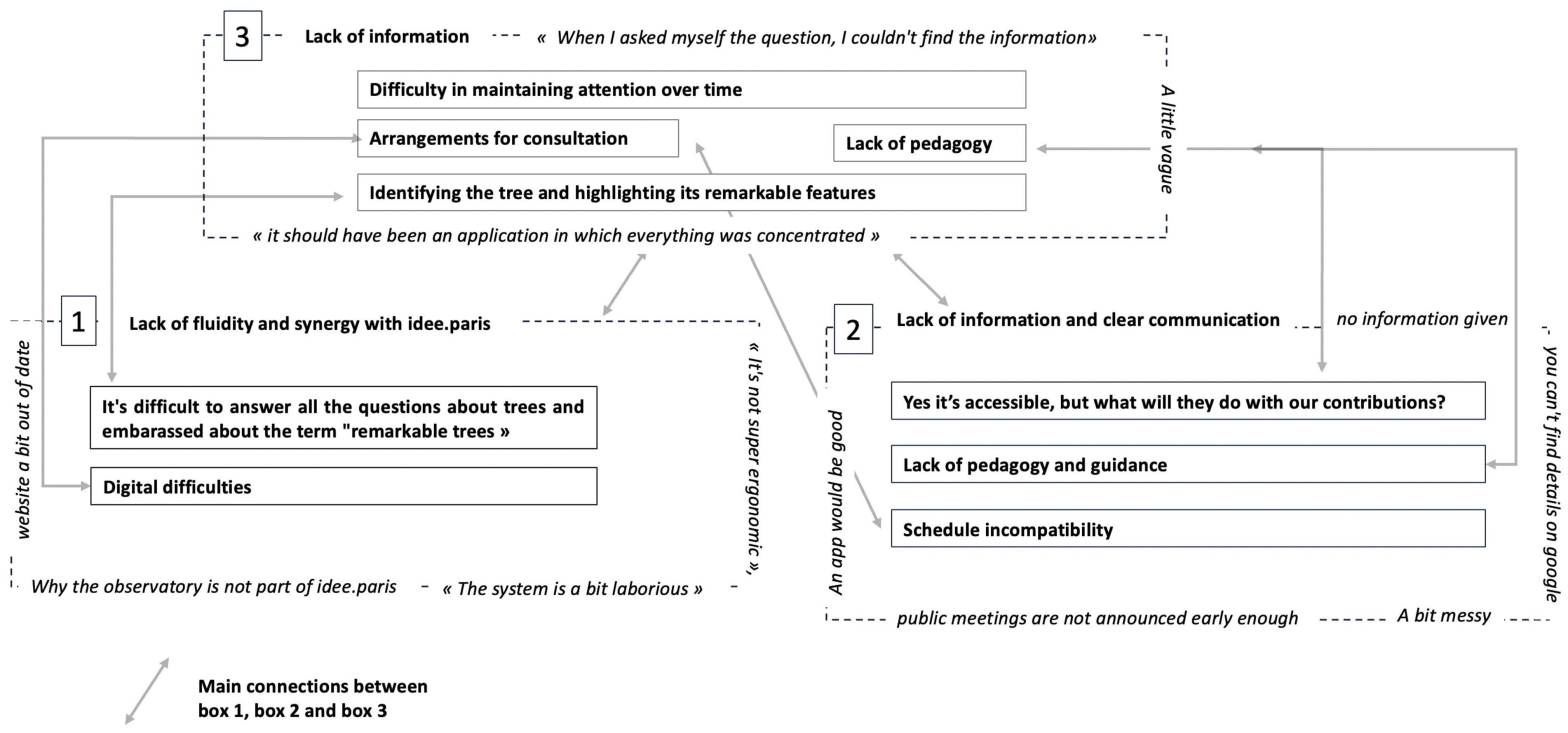
Obstacle connections.

*Lack of information and clear communication* is an obstacle that amplifies the others. *Lack of pedagogy and digital difficulties* are frequently mentioned and appear like strong obstacles to the 2 PS-CiTy success (referred to as *a societal problem, a brake on governance, and a lack of political attention*). Participants were also bothered by the term “remarkable” to qualify trees (*Biophilia and Urban Ecology*) (Figure 12).

**Figure 13:**
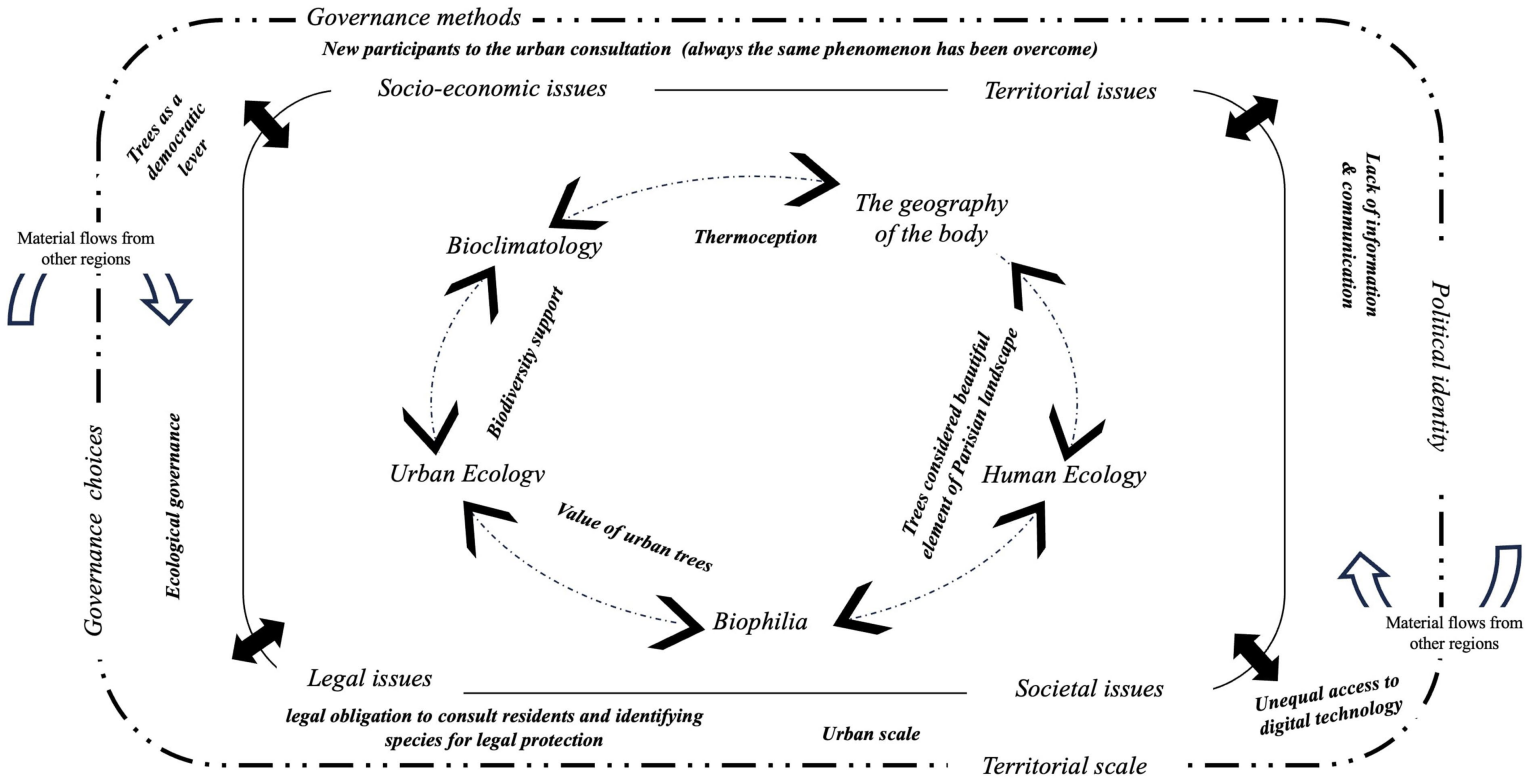
UPE theoretical framework applied to the 2 PS-CiTy.

As Figure 13 shows, the 2 PS-CiTy pilot project provides original results. It revealed the socio-ecological dynamics involved in urban trees to become levers for citizen engagement in urban consultation and their obstacles related to the political and societal context (Figure 13).

Nonetheless, this study did not permit to explore some of the UPE frame dimensions, such as “the non-hierarchical network of power extending beyond city and national boundaries (e.g., business networks, international clubs with local chapters, city partnerships, and transnational NGOs) that may affect local government and urban socionatures” (Cornea et al., 2017, p. 7). It would be relevant for another study to add results from interviews with a wider range of governance actors.

### 4.3 Suggestion to improve the 2 PS-CiTy

Our first suggestion to improve the 2 PS-CiTy is to underline the preservation of trees’ ecosystem services. Indeed, based on our result, we formulated a new hypothesis for a future research study that adding a census of ecosystem services provided by trees in the process would make the process more engaging (Table 2). Moreover, as part of a legal request for a building ban to preserve urban nature and considering the jurisprudence of the Council of State previously explained, this new census would also contribute to arguing that trees contribute to an essential territorial ecological objective for city dwellers’ wellbeing.

**Table 2:** The 2P.S-CiTy 3-stage process according to commit components.

5-stage process		The motivational component “why am I committing myself”	The behavioral component “How can I commit to?”	The cognitive component “What are the main goals of this commitment”
Stage 1	Questionnaire A *Parisian trees, the local urban plan and you*	**Identifying all kind of trees [not only those remarkable to me] motivates me;** **Specify the ecosystem service it provides to me while identifying the tree so that I can also report the tree’s ecosystem services;** Contributing to the preservation of trees in Paris encourages me to participate in the consultation; **Participating in a collective activity motivates me**	Even if it takes me some time, participating in this participatory process allows participants to be informed and express themselves; To participate in the consultation process related to the local urban plan with the same tools as the participatory programs and in the same framework to facilitate the process Despite a busy personal and professional schedule, I take the time to come to the participatory workshop; **The fact that the tree census and the urban consultation are taking place at the same time makes it easier for me to organize;** **Despite the technical nature of the LUP, I take the time to listen the presentation during the workshop.** Even if it requires time, I want to learn more and I read the documents relating to the Local Urban Plan; **Through sharing with others, I can overcome the difficulties I have with digital devices and ask for help**	To improve knowledge on nature in my town and on participatory processes; **To improve my knowledge on urban planning issues in my town;** **I understand the concrete positive effects of having identified a tree for preservation;** **To listen to the experts and decision makers explanation even if there is a disagreement between some participants because it brings elements of understanding and nourishes the dialogue;** **Share with others their experience of the walk and learn about others’ perceptions of the ecological services provided by trees;**
Stage 2	**To participate in a collective participatory workshop within the urban consultation of the LUP with an urban sensitivity walk to take pictures of trees and share your discovery, and the ecological services of trees that you perceived during the census.**			
Stage 3	A 30-min interview with A. Dakouré	Helping researchers explore new green and urban governance solutions by sharing my experience of the 2 PS-CiTy process motivates me	Even if it takes me some time, participating in this participatory process allows me to be informed and express myself	Thanks to the exchanges with the researchers, I understand more precisely the way in which the results will be represented, and I can ask to be notified of publications; I could share my feedback for them to improve the 2 PS-CiTy

The main changes made … in bold.

To complete the list of trees’ ecosystem services perceived by the respondents and to overcome the limits listed, we suggest organizing an in-person workshop to overcome digital difficulties, guide new respondents, and consolidate the community as soon as possible. This in-person workshop represents an opportunity to help identify tree species and ecosystem services (real and perceived), explain the Local Urban Plan perimeter, and connect the importance of tree census with the Local Urban Plan.

More precisely, we recommend organizing, during an in-person collective participatory workshop, an urban sensitivity walk within urban consultation of the LUP and adapting a pedagogical approach by inviting experts. The collective dimension of the workshop could allow respondents to share their perceptions and be positively influenced by the perceptions and knowledge of others.

As perceptions play an important role in the relationship with trees and the respondents’ perceptions are marked by ecosystem services, an urban sensory walk around trees could awaken new perceptions and complete the list of services provided by trees.

By focusing on ecosystem services provided by trees, this new version of the 2 PS-CiTy would directly relate trees to their urban environment, which could help respondents to associate trees with other urban issues during urban consultation.

This configuration reduces the number of steps from 5 to 3. We then suggest a 2 PS-CiTy process update based on the commit scale (Table 2).

Thus, for future pilot studies on this topic, we suggest starting from this new study framework (Table 2). However, conducting this new version of the pilot project requires more people to be involved. Indeed, at least two people are needed: A facilitator, who is different from the researcher, would have to organize and conduct the workshop so that the researcher could observe the workshop and the walk.

Moreover, to determine the contribution of a similar pilot project in terms of participants’ relationship with trees, especially in megapolis such as Paris, it would be interesting to use a representative sample drawn at random. In the longer term, repeating the experiment over the years with the same participants would help determine changes in their relationships with trees and nature at large in their city.

## 5 Conclusion

To conclude this study, we outline that the results of the 2 PS-CiTy revealed socio-ecological mechanisms that fall within the UPE’s fields of study identified above.

Indeed, in our study, to become a democratic lever, trees are first perceived and represented by their action on the micro-climate. This is explained by thermoception, which reflects the human body’s reaction to environmental changes. They are then considered for their ecological role in relation to other species and their beauty. Thanks to this description, the trees are identified as levers for civic engagement. However, according to feedback from participants, the power of urban trees is limited by the political and societal Parisian context. Indeed, participants expressed discomfort about the lack of information, communication, and transparency regarding urban consultation. They also mentioned the difficulties relating to digital consultation. Not everyone is equal regarding digital technology, which creates inequality in access to consultation. These obstacles represent important political and societal issues.

As the UPE framework underlines (Figure 13), it is clear that to improve urban ecological governance, local authority must (i) improve the information distribution on urban consultation, decision-making procedures, and the transparency of the political choices made and (ii) remove the filters of participation (illustrated here by unequal access to the digital technology or schedule difficulties).

The advantage of this framework is that it identifies and situates, within socio-ecological dynamics, the pilot project governance system characteristics. This statement suggests that once the system has been improved, it will be possible to analyze and compare the 2P.S-CiTy 3-stage process or other new modes of governance using this theoretical framework (Figure 1).

In the age of territorial ecological governance, municipalities are multiplying the tools they use to achieve their goals. To evaluate those tools, as well as new experiments, we suggest that decision-makers and technicians use a UPE theoretical analysis grid to adopt a systemic socio-ecological vision of urban transformations.

## Data availability statement

The raw data supporting the conclusions of this article will be made available by the authors, without undue reservation.

## Author contributions

AD: Conceptualization, Data curation, Formal analysis, Funding acquisition, Investigation, Methodology, Writing – original draft. J-YG: Supervision, Writing – review & editing.

## Appendix 1: Questionnaire A *Parisians trees, the local urban plan and you*

Among the five situations listed below, which one(s) best correspond(s) to you?

- I have already participated in the life of my neighborhood/city through participation in consultation events, involvement in associations or the organization of events to animate the life of my neighborhood or my city
- I have already participated in a participatory science program (Birds of the Gardens, Wild in my Street or others)
- I was or am part of an association that aims to protect and/or defend the environment/nature
- This is the first time, within the framework of this study, that I participate in a participative science program (Tree Observatory)
- It is the first time, within the framework of this study, that I will be involved in a consultation process (consultation on the PLU)

In a few words, what is the purpose of a Local Urban Plan for you?

Did you know, before agreeing to participate in this study, that the Local Urban Plan of Paris is currently being revised?

Is the fact that this survey could contribute to the Local Urban Plan of Paris, which is currently under revision, the reason for your participation?

Do you appreciate the presence of trees in the city?

If you answered “it depends” to the previous question, please specify

In a few words, what is a remarkable tree according to you?

Do remarkable trees, according to your own definition above, have an equivalent value to historic buildings for you?

In your opinion, do trees have to be remarkable to be preserved in the city? Please explain your answer in a few words

Does the preservation of remarkable trees encourage you to participate in the consultation on the regulations of the Local Urban Plan which will take place from next September?

Do you have a memory of a particular tree that has aroused a particular emotion in your life (in Paris or elsewhere, in a fictional or real space, in your childhood or recently)? Tell us, in a few words, about this memory

How do you perceive, during the day, the trees in the Parisian neighborhoods that you frequent most often?

- They catch your eye immediately
- Through the smells they give off
- By hearing the noise of the branches
- Other
- If you have indicated “Other”, please specify

How do you perceive, at night, the trees in the Parisian neighborhoods that you visit most often?

- They catch your eye immediately
- Through the smells they give off
- Through the refreshment they give you
- By hearing the noise of the branches
- Other

If you have indicated “Other”, please specify your thoughts:

How do you think of trees in Paris?

- As a shelter
- As a risk for underground networks
- As a support for biodiversity
- As a danger in case of storm
- As a symbol of nature in the city
- As a symbol of freedom
- As a characteristic of the Parisian public space
- As an obstacle to move easily in the streets (narrow and/or wide) on foot
- As beautiful elements of the landscape
- As ugly elements of the landscape
- As a source of freshness
- As a cause of insecurity when clusters of trees are not lit at night
- Other
- If you indicated “Other”, please specify

In what situations would you be unwilling or unable to accept the presence of a tree near your home?

- Planted in front of your window, it blocks out light
- Planted in front of your window, it attracts insects or birds including crows, pigeons...
- The tree’s roots damage public equipment (e.g. subway networks) near your home
- The tree is not in good health and represents a risk
- Other
- If you have indicated “Other”, please specify

Sociological profile: gender, age, socio-professional categories

How long have you lived in Paris?

In which borough do you live?

How long have you lived in this borough?

Which Parisian neighborhoods do you visit most often (at least once a week)?

## Appendix 2: Questionnaire B *Tell us about your discovery during the census of trees that you considered as remarkable*

Have you ever taken part in a census of plant or animal species before?

How many trees have you photographed and indicated on the Paris Tree Observatory website?

Did you know the name of the tree species when you took the photo(s)?

If so, which species were they?

Did you look up the name(s) of the species photographed that you did not know before indicating them on the Paris Tree Observatory website?

How did you search for the name(s) of the species? If you found it, please specify the name(s) of the species identified.

Where are the identified tree(s) located? Please specify, in a few words, the address (even approximately), the location (building yard, street...) and what this location means to you (on the way to work, near your home...)

Why was this tree or these trees remarkable to you?

What did this experience (taking a picture of the tree, taking a census, etc.) bring to you personally?

Would you like to participate in the consultation on the regulations of the future bioclimatic Local Urban Plan of Paris? Please explain your answer in a few words

Would you like to continue to participate in naturalist data collection programmes such as the Paris Tree Observatory? Please explain your answer in a few words

Please specify your email address below, this will allow me to link your different answers to the previous questionnaires
